# A predictive nomogram for surgical site infection in patients who received clean orthopedic surgery: a retrospective study

**DOI:** 10.1186/s13018-023-04473-2

**Published:** 2024-01-05

**Authors:** Zhi Li, Lihua Song, Baoju Qin, Kun Li, Yingtao Shi, Hongqing Wang, Huiwang Wang, Nan Ma, Jinlong Li, Jitao Wang, Chaozheng Li

**Affiliations:** 1 Department of Infection Management, North China Healthcare Group Xingtai General Hospital, Xingtai, Hebei China; 2 Operating Room, Xingtai General Hospital of North China Medical and Health Group, Xingtai, Hebei China; 3 Department of Orthopedics, North China Healthcare Group Xingtai General Hospital, Xingtai, Hebei China; 4 Hebei Provincial Key Laboratory of Precision Medicine for Liver Cirrhosis and Portal Hypertension, Xingtai People’s Hospital of Hebei Medical University, Xingtai, Hebei China

**Keywords:** Elective clean orthopedic surgery, Surgical site infection, Nomogram, Prediction model

## Abstract

**Background:**

Surgical site infection (SSI) is a common and serious complication of elective clean orthopedic surgery that can lead to severe adverse outcomes. However, the prognostic efficacy of the current staging systems remains uncertain for patients undergoing elective aseptic orthopedic procedures. This study aimed to identify high-risk factors independently associated with SSI and develop a nomogram prediction model to accurately predict the occurrence of SSI.

**Methods:**

A total of 20,960 patients underwent elective clean orthopedic surgery in our hospital between January 2020 and December 2021, of whom 39 developed SSI; we selected all 39 patients with a postoperative diagnosis of SSI and 305 patients who did not develop postoperative SSI for the final analysis. The patients were randomly divided into training and validation cohorts in a 7:3 ratio. Univariate and multivariate logistic regression analyses were conducted in the training cohort to screen for independent risk factors of SSI, and a nomogram prediction model was developed. The predictive performance of the nomogram was compared with that of the National Nosocomial Infections Surveillance (NNIS) system. Decision curve analysis (DCA) was used to assess the clinical decision-making value of the nomogram.

**Results:**

The SSI incidence was 0.186%. Univariate and multivariate logistic regression analysis identified the American Society of Anesthesiology (ASA) class (odds ratio [OR] 1.564 [95% confidence interval (CI) 1.029–5.99, *P* = 0.046]), operative time (OR 1.003 [95% CI 1.006–1.019, *P* < 0.001]), and D-dimer level (OR 1.055 [95% CI 1.022–1.29, *P* = 0.046]) as risk factors for postoperative SSI. We constructed a nomogram prediction model based on these independent risk factors. In the training and validation cohorts, our predictive model had concordance indices (C-indices) of 0.777 (95% CI 0.672–0.882) and 0.732 (95% CI 0.603–0.861), respectively, both of which were superior to the C-indices of the NNIS system (0.668 and 0.543, respectively). Calibration curves and DCA confirmed that our nomogram model had good consistency and clinical predictive value, respectively.

**Conclusions:**

Operative time, ASA class, and D-dimer levels are important clinical predictive indicators of postoperative SSI in patients undergoing elective clean orthopedic surgery. The nomogram predictive model based on the three clinical features demonstrated strong predictive performance, calibration capabilities, and clinical decision-making abilities for SSI.

**Supplementary Information:**

The online version contains supplementary material available at 10.1186/s13018-023-04473-2.

## Background

Surgical site infections (SSIs) are postoperative infections encompassing the superficial, deep, and interstitial layers [1–3]. SSI is a common nosocomial infection, leading to extended patient hospitalization and imposing substantial burdens on patients [1, 4, 5]. According to a U.S. Centers for Disease Control and Prevention health care-associated infection (HAI) prevalence survey, nearly 600,000 cases of SSI occurred in the USA in 2011, making it the most common HAI [6]. It is estimated that approximately 5% of patients develop SSI during the perioperative period, which prolongs the average length of stay by more than 9 days and increases the risk of death by 11 times [1].

Notably, orthopedic patients have heightened susceptibility to SSI relative to other patients owing to the enduring presence of internal fixation and implant apparatus within the body [7, 8]. These components create conducive niches and substrates for pathogenic proliferation, consequently significantly elevating the risk of postoperative wound infections [9, 10]. When SSI occurs during joint implant surgery, the cost per treatment may exceed $90,000 [2, 11, 12]. However, approximately 55% of SSIs are preventable through proper implementation of evidence-based strategies, so timely preoperative detection of high-risk SSI patients is critical [13].

The National Nosocomial Infections Surveillance (NNIS) risk index [14, 15] is the prevailing clinical prognostic instrument for predicting overall SSI risk. The NNIS system employs three autonomous and equitably significant variables—the American Society of Anesthesiology (ASA) classification [16], surgical incision type, and operative duration—to predict SSI risk. However, the prognostic efficacy of the NNIS system remains uncertain with respect to the prediction of SSI risk in patients undergoing elective aseptic orthopedic procedures [17, 18]. Consequently, the formulation of a composite predictive model based on multiple preoperative clinical parameters is imperative to aid orthopedic practitioners in identifying candidates at high risk of SSI.

A nomogram is a straightforward instrument for clinical prognostication and is used to predict clinical outcomes [19]. Nomograms have extensive applications across domains, such as oncology [20], cardiovascular ailments [21], and other medical conditions [22, 23]. Currently, the existing studies only focus on a certain special population, such as HIV patients [24], or a certain type of orthopedic surgery, such as lumbar instrumentation surgery [25], total knee arthroplasty [26], and posterior cervical surgery [27]. To address this, this investigation aimed to identify contributory risk factors associated with SSI in a subset of patients undergoing elective aseptic orthopedic surgery. The primary objective was to construct a predictive nomogram model for SSI risk to facilitate the identification of patients at high-risk of SSI.

## Methods

### Study design

This retrospective study included data from patients who underwent elective orthopedic procedures between January 2020 and December 2021. The inclusion criteria were as follows: (1) elective clean orthopedic surgery according the Centers for Disease Control and Prevention Guideline for the Prevention of Surgical Site Infection [2], and (2) comprehensive perioperative clinical data. The exclusion criteria were as follows: (1) emergency surgery, (2) incisions not meeting type I criteria, (3) preoperative community-acquired infection, and (4) insufficient pertinent perioperative clinical data.

Patients diagnosed with SSI were exhaustively identified, and a subset of patients without SSI was randomly selected to constitute the control cohort. The entire cohort was subsequently divided into training and validation cohorts using randomized sampling. The study adhered to the principles of the Declaration of Helsinki. All clinical data were de-identified, and the requirement for informed consent was waived following ethical committee approval.

### Definition of SSI

In alignment with the directives delineated in the Centers for Disease Control and Prevention and the American Academy of Orthopedic Surgery guidelines for the prevention of SSI, the diagnosis of SSI was confirmed through meticulous postoperative surveillance and adept interpretation of laboratory findings by seasoned surgical practitioners [2, 28]. The detailed definition of SSI is that it occurs within 30 days after surgery or within 1 year after foreign body implantation, which occurs in the skin and subcutaneous tissue of the surgical incision, or in the deep soft tissue (deep fascia and muscles) related to the surgery, or surgery-related organ or cavity infections, include superficial incision infection, deep incision infection and organ cavity infection [2].

### Collection of clinical variables

Baseline patient data and perioperative outcomes were gathered by two investigators (L.Z and L.K). The collected information included demographic data, foundational laboratory parameters, surgery-associated parameters, and postoperative follow-up results. Demographic variables included age, gender, body mass index, concurrent medical conditions (e.g., hypertension, diabetes, and coronary artery disease), and the ASA class. All laboratory indices were assessed 1–3 days prior to the procedure, including creatinine, albumin, total bilirubin, direct bilirubin, alanine aminotransferase, aspartate aminotransferase, prothrombin time, red blood cell count, and platelet count, among others.

Surgery-related details included surgical category (spine, extremities, and joints), NNIS grade, operative duration, estimated blood loss, preoperative skin condition, preoperative antibiotic administration, utilization of drains, and indwelling devices. Subsequent outcomes during the postoperative follow-up encompassed the occurrence of SSI and the duration of hospital stay.

### Statistical analysis

In this study, SPSS 26.0 (SPSS Inc, Chicago, IL, USA) and R Language software (version 3.5.3, http://www.r-project.org/)) were used for statistical analysis of the clinical data of the enrolled patients. Qualitative variables are described as absolute frequencies and percentages and were compared using the Kruskal–Wallis rank sum test. Quantitative variables are expressed as mean ± standard deviation and compared using the Mann–Whitney U-test or Student’s t-test. Univariate logistic regression analysis was used to determine risk factors associated with SSI. Indices with *P* < 0.1 in the univariate analysis were included in the multivariate analysis. Based on the independent risk factors screened using the results of the multivariate analysis, a nomogram model was constructed using R software. The area under the receiver operating characteristic (ROC) curve, sensitivity, specificity, positive predictive value (PPV), negative predictive value (NPV), and other indicators were used to compare the predictive performances of the nomogram prediction model and the NNIS classification. The concordance index (C-index) and consistency calibration curves were used to analyze the predictive performance of the nomogram model. Decision curve analysis (DCA) was used to measure the clinical utility of the nomogram by calculating the net benefit of different threshold probabilities. *P* < 0.05 was considered statistically significant.

## Results

### Patient characteristics

A total of 20,960 patients underwent elective clean orthopedic surgery in our hospital between January 2020 and December 2021, of whom 39 developed SSI; the SSI incidence was 0.186%. All 39 patients with a postoperative diagnosis of SSI and a randomly selected 305 patients who did not develop SSI postoperatively were included in the final analysis. The median age of the 344 patients was 55.0 years (interquartile range, 40.8–66.0 years) and 159 (46.2%) of them were female. In the total cohort, 212 (61.6%) patients had comorbidities before surgery. Seventy seven (22.38%), 235 (68.31%), and 32 (9.3%) patients had ASA classes 1, 2, and 3, respectively. In the total cohort, 291 (84.59%) and 53 (15.41%) patients had NNIS grades 0 and 1, respectively. The total cohort was randomly divided into a training cohort (*n* = 240) and a validation cohort (*n* = 104) in a 7:3 ratio. The proportion of patients with SSI was not significantly different between the training and validation cohorts, and all baseline characteristics were comparable (*P* > 0.05). The baseline characteristics of the total, training, and validation cohorts are presented in Table 1. Additional file 1: Table S1 showed the differences in baseline information between the SSI and non-SSI groups.

**Table 1:** Characteristics of patients in the total, training, and validation cohorts

Characteristics	Total cohort (*n* = 344)	Training cohort (*n* = 240)	Validation cohort (*n* = 104)	*P* value
Age, year	55.0 (40.8–66.0)	54.0 (39.0–65.0)	59.5 (45.0–66.0)	0.269
*Gender*				0.490
Female	159 (46.2)	108 (40.8)	51 (49.0)	
Male	185 (53.8)	132(59.1)	53 (50.1)	
*Comorbidities*				0.982
No	132 (38.4)	92 (38.3)	40 (38.5)	
Yes	212 (61.6)	148 (61.7)	64 (61.5)	
BMI	24.9 (22.0–27.8)	24.7 (22.3–28.1)	25.0 (21.7–27.6)	0.554
*Surgical site*				0.458
Joint	96 (27.91)	64 (26.67)	32 (30.77)	
Spine	77 (22.38)	58 (24.17)	19 (18.27)	
Limbs	171 (49.71)	118 (49.17)	53 (50.96)	
*ASA class*				0.214
1	77 (22.38)	56 (23.33)	21 (20.19)	
2	235 (68.31)	166 (69.17)	69 (66.35)	
3	32 (9.3)	18 (7.5)	14 (13.46)	
*NNIS class*				0.519
0	291 (84.59)	205 (85.42)	86 (82.69)	
1	53 (15.41)	35 (14.58)	18 (17.31)	
*Postoperative drainage*				0.381
No	232 (67.44)	158 (65.83)	74 (71.15)	
Yes	112 (32.56)	82 (34.17)	30 (28.85)	
*Implants*				0.062
No	90 (26.16)	70 (29.17)	20 (19.23)	
Yes	254 (73.84)	170 (70.83)	84 (80.77)	
Operation time	67.4 (45.8–106.1)	67.6 (45.0–109.0)	67.2 (48.4–87.3)	0.791
Length of hospital stay	3.0 (2.0–5.0)	3.0 (2.0–5.0)	3.0 (2.0–5.0)	0.736
RBC, 1012 /L	4.4 (4.0–4.8)	4.5 (4.1–4.8)	4.4 (3.9–4.6)	0.122
WBC, 109 /L	6.6 (5.5–7.9)	6.4 (5.3–7.8)	6.9 (5.9–8.0)	0.124
Platelet, 109 /L	237.0 (196.0–282.5)	234.0 (196.0–282.5)	240.5 (199.0–282.8)	0.677
Haemoglobin, g/L	133.0 (120.0–144.2)	133.0 (121.0–146.0)	131.0 (117.5–142.2)	0.076
AST, U/L	20.0 (15.0–25.0)	19.0 (15.0–25.0)	20.0 (16.0–24.0)	0.361
ALT, U/L	18.0 (13.0–27.0)	18.0 (13.0–27.0)	18.5 (13.0–26.2)	0.584
Total bilirubin, μmol/L	15.3 (11.8–19.1)	15.3 (11.8–19.2)	15.3 (11.3–18.9)	0.699
Direct bilirubin, μmol/L	3.6 (2.8–4.8)	3.6 (2.8–4.9)	3.6 (2.9–4.8)	0.714
GGT, IU/L	20.0 (17.0–24.0)	20.0 (17.0–23.2)	20.0 (17.8–24.2)	0.947
Albumin, g/L	40.7 (39.0–42.8)	40.7 (39.1–42.8)	40.7 (38.1–42.1)	0.078
Creatinine, μmol/L	58.5 (51.9–68.1)	58.5 (51.9–68.3)	58.3 (52.1–67.3)	0.887
Prothrombin time, s	12.1 (11.3–12.7)	12.1 (11.3–12.8)	12.0 (11.3–12.5)	0.206
D-Dimer, μg/mL	0.5 (0.2–1.1)	0.5 (0.2–1.1)	0.5 (0.2–1.5)	0.464
*SSI*				0.999
No	305 (88.66)	213 (88.75)	92 (88.46)	
Yes	39 (11.34)	27 (11.25)	12 (11.54)	

*ASA* American society of anesthesiology, *NNIS* national nosocomial infections surveillance, *RBC* Red blood cell, *WBC* white blood cell, *ALT* alanine aminotransferase, *AST* aspartate aminotransferase, *BMI* body mass index, *GGT* gamma-glutamyl transferase, *SSI* surgical site infection

### Univariate and multivariate analyses of risk factors for SSI

Univariate analysis confirmed that the ASA class (odds ratio [OR] 2.242 [95% confidence interval (CI) 1.035–5.03, *P* = 0.045]), length of hospital stay (OR 1.014 [95% CI 1.008–1.02, *P* < 0.001]), surgery duration (OR 1.171 [95% CI 1.041–1.333, *P* = 0.011]), and D-dimer level (OR 1.169 [95% CI 1.049–1.386, *P* = 0.033]) were significant predictors of SSI occurrence in these patients (Table 2).

**Table 2:** Univariate and multivariate analyses of factors associated with surgical site infection

Characteristics	Univariate analysis		Multivariate analysis	
	OR (95% CI)	*P* value	OR (95% CI)	*P* value
(Intercept)			3.008 (0–0.0230)	< 0.001
Age, year	0.993 (0.973–1.014)	0.497		
BMI	1.076 (0.997–1.160)	0.055		
ASA class	2.242 (1.035–5.030)	0.045	1.564 (1.029–5.990)	0.046
NNIS class	6.609 (2.733–15.904)	< 0.001		
Postoperative drainage	4.656 (2.033–11.387)	< 0.001		
Implants	2.582 (0.947–9.065)	0.091		
Operation time	1.014 (1.008–1.020)	< 0.001	1.003 (1.006–1.019)	< 0.001
Length of hospital stay	1.171 (1.041–1.333)	0.011	1.065 (0.969–1.258)	0.101
RBC, 10^12^ /L	0.872 (0.438–1.750)	0.698		
WBC, 10^9^ /L	1.177 (0.997–1.382)	0.048		
Platelet, 10^9^ /L	1.001 (0.996–1.005)	0.685		
Haemoglobin, g/L	0.999 (0.977–1.021)	0.906		
AST, U/L	1.018 (0.994–1.041)	0.108		
ALT, U/L	1.003 (0.982–1.019)	0.742		
Total bilirubin, μmol/L	1.033 (0.985–1.079)	0.148		
Direct bilirubin, μmol/L	1.109 (0.947–1.274)	0.162		
GGT, IU/L	0.998 (0.976–1.011)	0.842		
Albumin, g/L	0.976 (0.872–1.096)	0.676		
Creatinine, μmol/L	0.991 (0.961–1.019)	0.532		
Prothrombin time, s	0.964 (0.623–1.486)	0.866		
D-Dimer, μg/mL	1.169 (1.049–1.386)	0.033	1.055 (1.022–1.29)	0.046

*ASA* American society of anesthesiology, *NNIS* national nosocomial infections surveillance, *RBC* Red blood cell, *WBC* white blood cell, *ALT* alanine aminotransferase, *AST* aspartate aminotransferase, *BMI* body mass index, *GGT* gamma-glutamyl transferase

We incorporated all risk factors identified by univariate analysis (*P* < 0.1) into a multivariate logistic regression model to predict the significant independent risk factors. Multivariate logistic regression analysis showed that the risk factors for postoperative SSI included ASA class (OR 1.564, [95% CI 1.029–5.99, *P* = 0.046]), surgery duration (OR 1.003 [95% CI 1.006–1.019, *P* < 0.001]), and D-dimer level (OR 1.055 [95% CI 1.022–1.29, *P* = 0.046]) (Table 2).

### Nomogram for predicting SSI

**Fig. 1:**
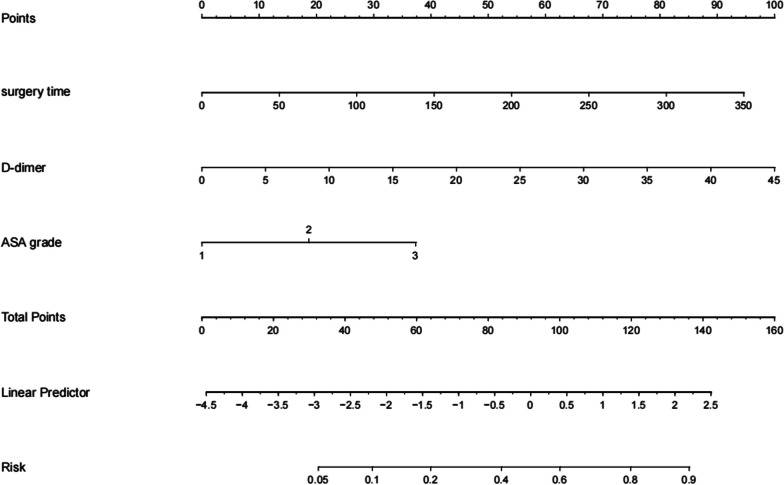
A nomogram model for predicting overall risk of surgical site infection after elective orthopedic surgery

The independent factors identified in the multivariate analysis (surgery duration, ASA class, and D-dimer level) were included in the nomogram predictive model of SSI occurrence using the RMS package in the R software. The total score was calculated by assigning values to the three clinical features. The linear predictor value was obtained from the caliper, and the probability of SSI occurrence was determined (Fig. 1).

We further developed a web calculator based on the nomogram prediction model for clinicians and researchers to predict patients’ SSI probability by simply entering three clinical characteristics (https://jitao.shinyapps.io/dynnomapp/). Using a formula based on our nomogram model, the probability of SSI for patient 3 in the validation cohort was calculated to be 24%, which is close to the result of the web calculator (25%, 95% CI 16.3–36.4), validating the calculator accuracy (Additional file 1: Fig. S1).

### Predictive value of the nomogram model for SSI in the training group

**Fig. 2:**
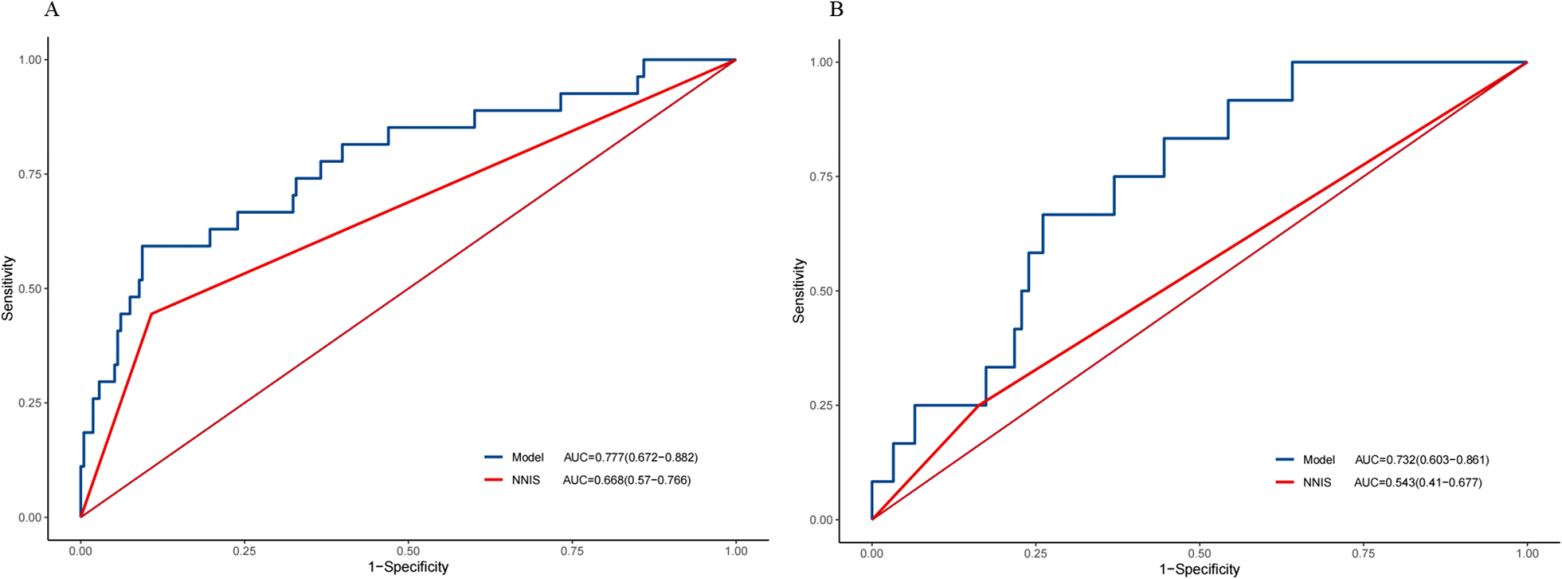
ROC curve analysis was used to compare the performance of the nomogram and the NNIS system for predicting surgical site infection in **A** the training cohort and **B** the validation cohort. *ROC* receiver operating characteristic, *NNIS* national nosocomial infections surveillance

**Fig. 3:**
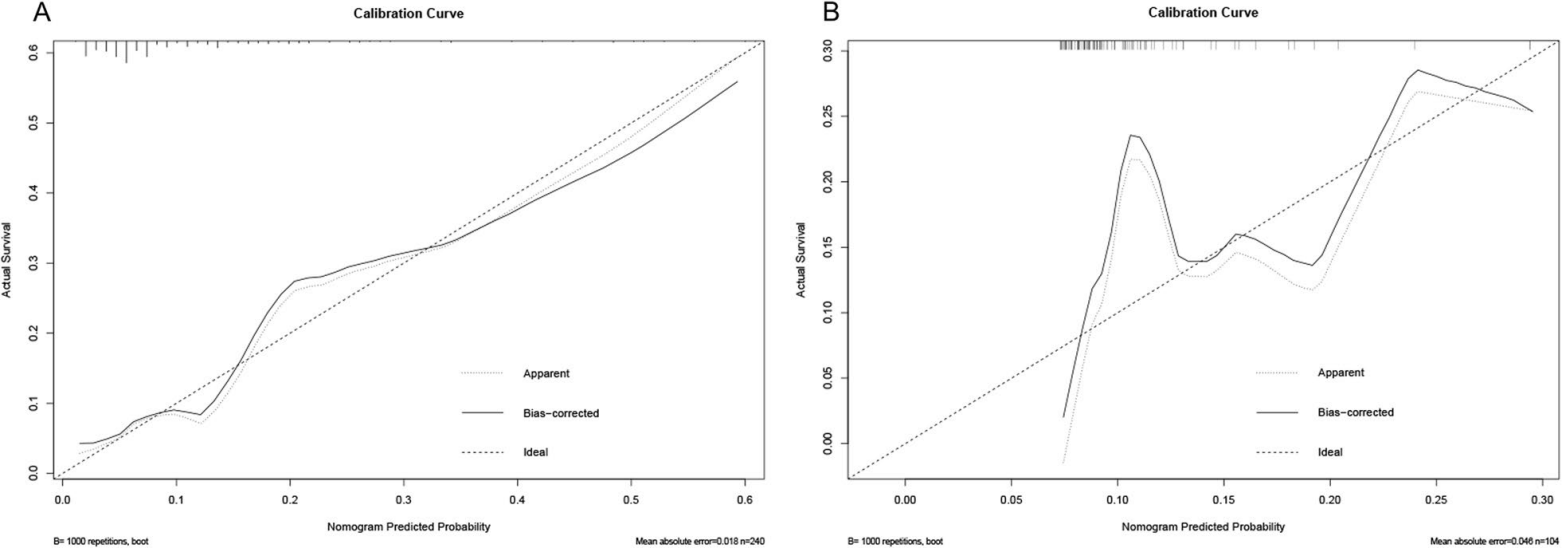
Calibration curves of the nomogram for predicting the risk of surgical site infection in **A** the training cohort and **B** the validation cohort

The C-index of our nomogram was 0.777 (95% CI 0.672–0.882), which was significantly higher than the NNIS grading C-index of 0.668 (95% CI 0.570–0.766) (Fig. 2A). The calibration curve of the SSI prediction showed that the nomogram-predicted likelihood of developing SSI was in good agreement with the observed development of SSI (Fig. 3A).

### Predictive value of the nomogram model for SSI in the validation cohort

The nomogram also showed higher performance in predicting SSI in the validation cohort, with a C-index of 0.732 (95% CI 0.603–0.861), compared with the C-index of 0.543 (95% CI 0.410–0.677) for the NNIS system (Fig. 2B). In addition, the calibration curve of the SSI forecast showed that the nomogram agreed well with the observed development of SSI. This demonstrates that our nomogram-based prediction model had good predictive performance for the occurrence of SSI in the validation cohort (Fig. 3B).

### Comparison of the nomogram and NNIS system

In the training cohort, the nomogram showed favorable predictive performance for SSI detection, with an NPV, PPV, specificity, sensitivity, accuracy, precision, and recall of 0.946, 0.444, 0.906, 0.593, 0.871, 0.444, and 0.593, respectively (Table 3). The nomogram had a higher predictive performance for SSI than the NNIS system (Table 3). The nomogram also showed good performance for SSI in the validation cohort (Table 4). The NPV, PPV, specificity, sensitivity, accuracy, precision, and recall for the nomogram and NNIS system in the validation cohort are summarized in Table 4.

**Table 3:** Performance of the nomogram model and NNIS system for predicting surgical site infection in the training cohort

	Negative predictive value	Positive predictive value	Specificity	Sensitivity	Accuracy	Precision	Recall
Nomogram model	0.946	0.444	0.906	0.593	0.871	0.444	0.593
NNIS system	0.927	0.343	0.892	0.444	0.842	0.343	0.444

*NNIS* national nosocomial infections surveillance

**Table 4:** Performance of the nomogram model and NNIS system for predicting surgical site infection in the validation cohort

	Negative predictive value	Positive predictive value	Specificity	Sensitivity	Accuracy	Precision	Recall
Nomogram model	0.944	0.250	0.739	0.667	0.731	0.250	0.667
NNIS system	0.895	0.167	0.837	0.250	0.769	0.167	0.250

*NNIS* national nosocomial infections surveillance

**Fig. 4:**
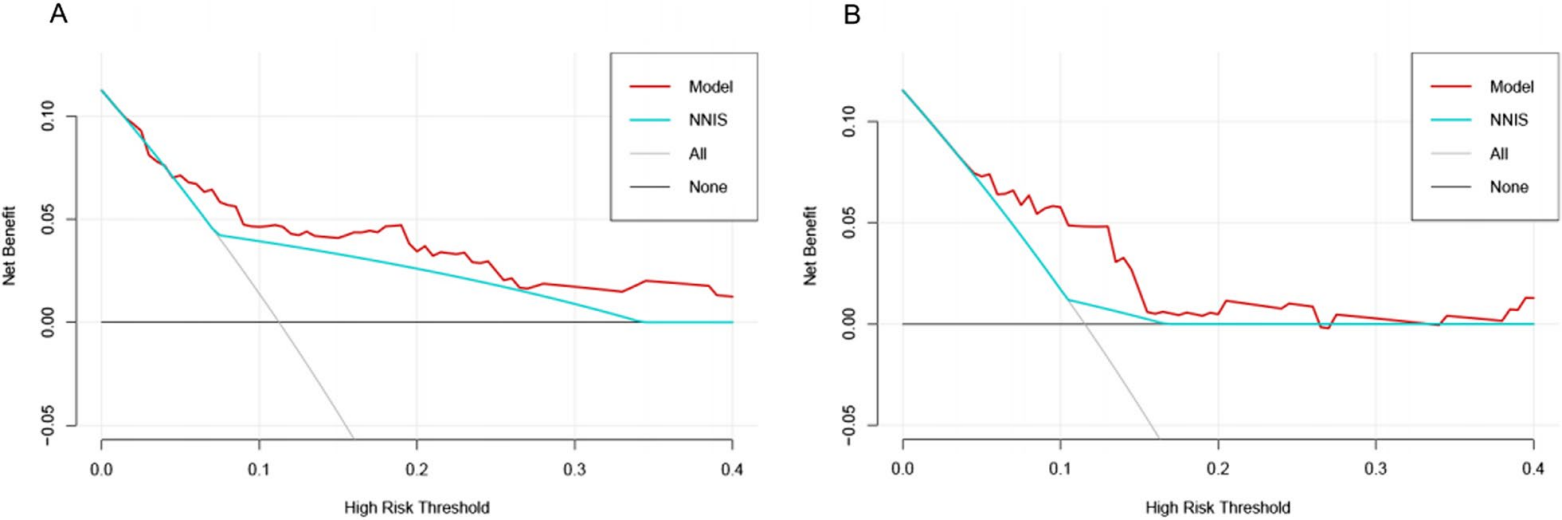
Decision curve analysis for the nomogram and the NNIS system for surgical site infection in **A** the training cohort and **B** the validation cohort

DCA showed that the nomogram for predicting SSI was more valuable than the NNIS system in the training cohort (Fig. 4A) and in the validation cohort (Fig. 4B). Therefore, our nomogram outperforms the existing models.

## Discussion

Despite advances in the management of perioperative nosocomial infections in recent years, SSIs remain a common cause of increased mortality, length of stay, and cost in surgical patients [1, 2]. Our investigation devised a model aimed at predicting the incidence of SSI in individuals undergoing clean orthopedic surgery, thereby proficiently evaluating the risk of SSI among elective aseptic orthopedic patients. Using univariate and multivariate logistic regression analyses, we established that operation time, ASA class, and D-dimer level were independently correlated with a heightened risk of postoperative SSI. Subsequently, the logistic regression model was translated into a visual representation a nomogram. Our nomogram model not only exhibited robust predictive capability and impeccable calibration but also had substantial clinical utility in facilitating informed decision-making for patients within both the training and validation cohorts. Additionally, we extended our efforts to develop an easy-to-use and free-to-access online calculator based on the nomogram model (https://jitao.shinyapps.io/dynnomapp/), an accessible tool designed to enable clinicians and researchers to readily ascertain the probability of postoperative SSI in the special patient populations.

The American College of Surgeons incision grading system stratifies incisions into four distinct grades: grade I, grade II, grade III, and grade IV. Grade I incisions, characterized as clean surgeries, exhibit a propensity for swift and comprehensive healing within a condensed timeframe. Directives formulated by the US Centers for Disease Control and Prevention state that clean surgeries, including of drainage procedures, require no supplementary antibiotic prophylaxis after closure of the surgical incision [2]. Although, compared with other types of surgery, the risk of SSI in patients undergoing clean orthopedic surgery is relatively low, once SSI occurs, it may lead to serious clinical outcome [29, 30].

The NNIS grading system is currently the most widely used clinical tool for predicting the occurrence of SSI and includes three independent and equally important variables: ASA class, surgical incision type, and surgical duration. Through the qualitative classification of these variables, the NNIS system divides the surgical risk into four levels, namely, NNIS level 0, NNIS level 1, NNIS level 2, and NNIS level 3 [14, 15]. However, because all surgical incision types in clean surgery are the same, the NNIS system lacks specificity for clean surgery. Compared to the NNIS system, our nomogram model integrates qualitative and quantitative clinical variables. By assigning values to each clinical variable and intuitively obtaining the occurrence probability of SSI with a 95% CI, the nomogram is more convenient for orthopedic surgeons. More importantly, our predictive model had a higher predictive ability and is more suitable than traditional NNIS system for patients undergoing clean orthopedic surgery.

Similar to the NNIS system, our nomogram included the ASA class and operation time, as they were independent risk variables for SSI. ASA classification is a clinical tool used to assess the risk of developing SSI and severity of potential disease in patients undergoing preoperative anesthesia. Many studies have confirmed that ASA classification can be used for SSI risk stratification [31–34]. A study of 310 patients who underwent general surgery and were classified as clean or clean-contaminated confirmed that the rate of SSI was significantly higher in patients with ASA class II-III than in patients with ASA class I (*P* = 0.003). An ASA class > 2 is independently associated with SSI [33]. The duration of surgery is another widely recognized clinical index closely related to the occurrence of SSI. In a study of 825 patients undergoing spinal surgery, operative time (*P* = 0.0019) and ASA class III (*P* = 0.0132) were independent risk factors for SSI [32]. Higher ASA classes are associated with more comorbidities and poorer immunity, whereas longer operation time usually indicates higher surgical difficulty and longer incision exposure time, all of which increase the risk of pathogen invasion [32, 35, 36]. Therefore, shortening the operation time, especially in patients with higher ASA classes, can effectively prevent SSI.

Our predictive model also incorporates another laboratory measure, the D-dimer level, which is not included in the NNIS system. Owing to the close relationship between the coagulation system, inflammation, and endothelial injury, an increase in D-dimer levels is also often observed in some non-thrombotic diseases, such as infection, surgery, trauma, heart failure, and malignant tumors [37–39]. A multicenter study of patients undergoing revision total joint arthroplasty examined elevated serum C-reactive protein (CRP > 1 mg/dL), D-dimer (> 860 ng/mL), and erythrocyte sedimentation rate (> 30 mm/h), which were assigned 2, 2, and 1 points, respectively, and jointly constructed a new standard for the diagnosis of periprosthetic infection (PJI) with other laboratory indicators; its sensitivity and specificity were significantly higher than those of the Musculoskeletal Infection Association and International Consensus Conference Definition [40]. Another study demonstrated that a serum D-dimer threshold of 0.75 mg/L predicted shoulder PJI with a sensitivity of 86%, specificity of 56%, and area under the curve of 0.74. When serum D-dimer and CRP above thresholds of 0.75 mg/L and 10 mg/L, respectively, were used to predict PJI, the sensitivity and specificity were 57% and 100%, respectively [41]. Therefore, it is necessary to maintain D-dimer levels in patients at normal or even slightly decreased levels to reduce the incidence of SSI [41–44].

This study had some limitations. First, owing to the retrospective nature of the study, it only included a small number of patients who did not develop SSI, and selection bias was inevitable. Second, some inflammatory indicators that may be related to SSI, such as C-reactive protein and procalcitonin, were missing from our study; the inclusion of these indicators may help improve the predictive accuracy of the model. Third, this was a single-center study. To verify the prediction model, we randomly divided the total cohort into training and internal validation cohorts; however, we still lacked an external validation cohort. In the future, another prospective multicenter study with a larger sample size is needed to further confirm the predictive performance of this model. Finally, models based on more advanced machine learning algorithms or radiomics may be more helpful in providing predictive model accuracy [45–47]. Further development of SSI models based on other artificial intelligence is still needed to further improve prediction capabilities.

## Conclusions

In conclusion, operation time, ASA class, and D-dimer level are important clinical indicators of postoperative SSI in patients undergoing elective clean orthopedic surgery. The nomogram prediction model based on these clinical characteristics showed strong SSI prediction performance, calibration, and clinical decision-making utility. In addition, we created an online calculator using the nomogram so that orthopedic surgeons and researchers can easily and quickly predict the risk of postoperative SSI and identify patients at high risk as early as possible to reduce the risk of infection.

### Supplementary Information

**Additional file 1.** **Supplement Table 1.** Characteristics of patients in the Non-SSI group and the SSI group.

## Data Availability

The data of this study are available from the corresponding author upon request.

## Abbreviations

ASA: American society of anesthesiology
CI: Confidence interval
C-index: Concordance-index
CRP: C-reactive protein
DCA: Decision curve analysis
NNIS: National nosocomial infections surveillance
NPV: Negative predictive value
OR: Odds ratio
PJI: Periprosthetic infection
PPV: Positive predictive value
SSI: Surgical site infection
