# Supplementary material for: A predictive nomogram for surgical site infection in patients who received clean orthopedic surgery: a retrospective study
Source: J Orthop Surg Res. 2024 Jan 5;19:38. doi: 10.1186/s13018-023-04473-2 (PMC10770936; doi:10.1186/s13018-023-04473-2)
Supplement: Supplementary file 1 — Additional file 1. Supplement Table 1. Characteristics of patients in the Non-SSI group and the SSI group. [file 13018_2023_4473_MOESM1_ESM.docx]

Supplementary Material

**Supplement Table 1 Characteristics of patients in the Non-SSI group and the SSI group**

| Characteristics | **Non-SSI group**  **(n = 305)** | **SSI group**  **(n = 39)** | P-value |
| --- | --- | --- | --- |
| Age-year | 55 (40 - 66) | 52 (41.5 - 65) | 0.348 |
| BMI | 24.8 (22.2- 27.7) | 25.39 (21.4- 29.6) | 0.548 |
| Surgical site |  |  | 0.099 |
| Joint | 87 (29) | 9 (23) |  |
| Spine | 63 (21) | 14 (36) |  |
| Limbs | 155 (51) | 16 (41) |  |
| ASA class |  |  | 0.036 |
| 1 | 74 (24) | 3 (8) |  |
| 2 | 204 (67) | 31 (79) |  |
| 3 | 27 (9) | 5 (13) |  |
| NNIS class |  |  | < 0.001 |
| 0 | 267 (88) | 24 (62) |  |
| 1 | 38 (12) | 15 (38) |  |
| Postoperative drainage |  |  | < 0.001 |
| No | 217 (71) | 15 (38) |  |
| Yes | 88 (29) | 24 (62) |  |
| Implants |  |  | 0.027 |
| No | 86 (28) | 4 (10) |  |
| Yes | 219 (72) | 35 (90) |  |
| Operation time | 64.55 (44.0- 95.5) | 121.45 (67.6- 163.3) | < 0.001 |
| Length of hospital stay | 3 (2- 4) | 3 (2- 7) | 0.173 |
| RBC- 1012 /L | 4.48 (4.0 - 4.8) | 4.29 (4.1 - 4.6) | 0.212 |
| WBC- 109 /L | 6.59 (5.5 - 7.9) | 7.36 (5.5 - 9.0) | 0.103 |
| Platelet- 109 /L | 238 (196- 281) | 232 (191 - 303.5) | 0.911 |
| Haemoglobin- g/L | 133 (120 - 145) | 133 (122 - 141) | 0.51 |
| AST- U/L | 20 (16 - 24) | 20 (15- 25) | 0.742 |
| ALT- U/L | 18 (13 - 26) | 18 (12.5- 31) | 0.994 |
| Total bilirubin- μmol/L | 15.3 (11.6- 19.0) | 16.5 (12.1- 20.2) | 0.417 |
| Direct bilirubin- μmol/L | 3.6 (2.8 - 4.8) | 3.6 (2.9 - 4.75) | 0.693 |
| GGT- IU/L | 20 (17 - 24) | 20 (18 - 20) | 0.393 |
| Albumin- g/L | 40.7 (39 - 42.9) | 40.2 (38.6 - 41.4) | 0.092 |
| Creatinine- μmol/L | 58.5 (51.9 - 68.2) | 56.5 (52.2 - 65.) | 0.739 |
| Prothrombin time- s | 12.02 ± 0.93 | 12 ± 1.1 | 0.943 |
| D-Dimer- μg/mL | 0.46 (0.21 - 1.03) | 0.61 (0.31 - 3.05) | 0.036 |

SSI:surgical site infection; ASA: American Society of Anesthesiology; NNIS: National Nosocomial Infections Surveillance; RBC: Red blood cell; WBC: White blood cell;ALT: Alanine aminotransferase; AST: Aspartate aminotransferase; BMI: Body mass index; GGT:gamma-glutamyl transferase.

## Supplementary Figure

**Supplementary Figure 1.** The web-based calculator for predicting surgical site infection probability of patients who received clean orthopedic surgery.

**
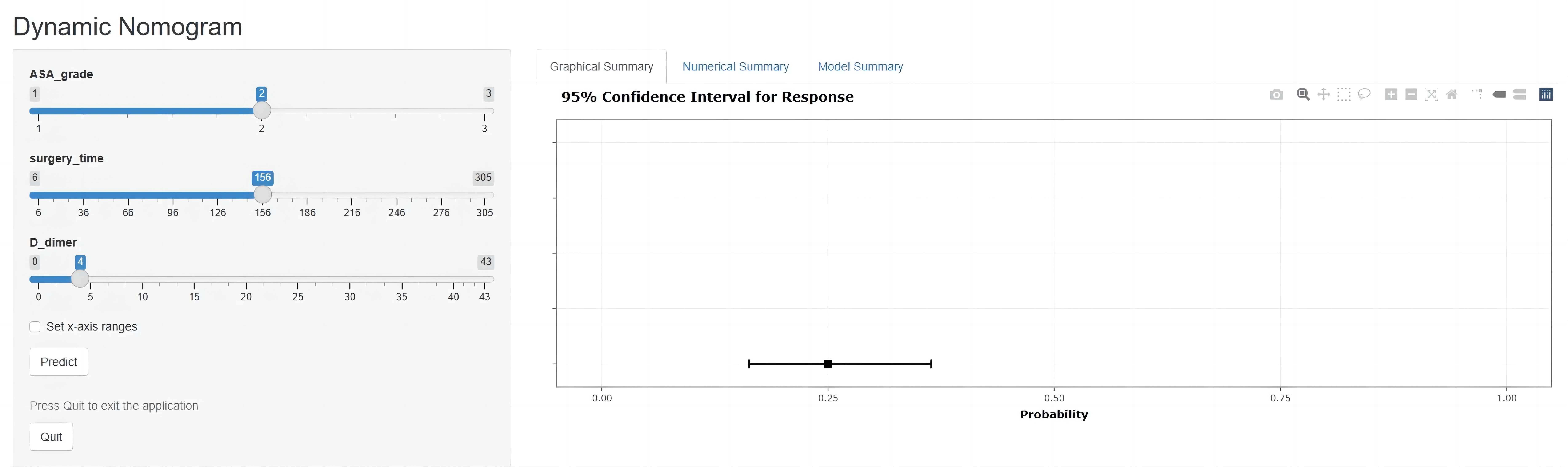
**
